# Unveiling Lipid Metabolism-Related Gene PTGDS: A Tumor Suppressor in Lung Adenocarcinoma with Therapeutic Potential

**DOI:** 10.3390/cancers18121884

**Published:** 2026-06-09

**Authors:** Boxuan Zhou, Jianwei Shi, Linchuan Liang, Yushun Gao

**Affiliations:** Department of Thoracic Surgery, National Cancer Center/National Clinical Research Center for Cancer/Cancer Hospital, Chinese Academy of Medical Sciences and Peking Union Medical College, Chaoyang District, Panjiayuan, Nanli 17, Beijing 100021, China; bxzhoupumc@163.com (B.Z.); syxxsjw@163.com (J.S.);

**Keywords:** cell cycle, immune cell infiltration, lipid metabolism, lung adenocarcinoma, prognostic biomarker, PTGDS

## Abstract

Background: Lung adenocarcinoma (LUAD) remains a leading cause of cancer-related mortality worldwide. Although the lipid metabolism-associated gene PTGDS has been implicated in tumorigenesis, its functional significance and regulatory mechanisms in LUAD are poorly understood. Methods: We integrated multi-omics data from TCGA and GEO cohorts to evaluate PTGDS expression and its clinicopathological relevance. Functional characterization was performed using gain-of-function models in LUAD cell lines and a xenograft mouse model, assessing proliferation, migration, invasion, and immune microenvironment alterations. Results: PTGDS expression is markedly reduced in LUAD tissues and correlates strongly with advanced disease stage and unfavorable prognosis. Clinically, low PTGDS expression is associated with specific driver mutations and reduced tumor mutation burden. Notably, PTGDS levels correlate with enhanced cytotoxic T-cell infiltration and suppressed M2 macrophage polarization, suggesting immunomodulatory functions. Ectopic expression of PTGDS significantly suppressed malignant behaviors in vitro and tumor growth in vivo. Mechanistically, PTGDS overexpression was associated with reduced expression of CDK1 and PLK1 and increased expression of p21 and p27. Conclusions: Our findings establish PTGDS as a novel tumor suppressor in LUAD that restrains tumor progression through cell cycle modulation and immune microenvironment remodeling, highlighting its potential as both a prognostic biomarker and a therapeutic target.

## Simple Summary

Lung adenocarcinoma (LUAD) is a common and aggressive lung cancer. New methods to predict patient outcomes and treat the disease are urgently needed. In this study, we focused on a lipid metabolism-related gene, PTGDS. By analyzing large cancer databases and performing experiments in cells and mice, we found that PTGDS levels are much lower in LUAD tumors than in healthy lung tissue. Patients with lower PTGDS levels tended to have more advanced cancer and shorter survival. Increasing PTGDS in cancer cells slowed tumor growth, migration, and invasion, while reducing PTGDS had the opposite effects. PTGDS also affected key cell cycle proteins, including CDK1, PLK1, p21, and p27. Computer-based analyses suggested a link between PTGDS and immune cell activity, though this requires experimental confirmation. Together, our results indicate that PTGDS acts as a natural brake on LUAD progression and could serve as a new prognostic biomarker and potential therapeutic target.

## 1. Introduction

Lung cancer is the most common malignancy globally [1], responsible for approximately 18% of cancer-related deaths and representing one of the leading causes of cancer mortality [2]. Lung adenocarcinoma (LUAD) and squamous cell carcinoma (LUSC) are the predominant subtypes of non-small cell lung cancer (NSCLC), accounting for approximately 82% of all lung cancer cases [3]. LUAD, a major subtype of NSCLC, constitutes approximately 40% of lung cancer cases [4]. The overall five-year survival rate for patients with LUAD remains poor despite substantial progress in diagnostic and therapeutic approaches for lung cancer over the past decade, particularly for those with late-stage disease, post-treatment recurrence, and metastasis [5,6]. Therefore, it is crucial to investigate the underlying molecular mechanisms driving LUAD development and to identify potential therapeutic targets.

Metabolic dysregulation is a hallmark of cancer that allows tumor cells to adapt to their specific energy requirements through metabolic reprogramming. Lipid metabolic reprogramming is a common feature across nearly all types of cancer. Studies have confirmed that metabolic reprogramming is closely associated with tumor initiation, progression, metastasis, remodeling of the immune microenvironment, and chemoresistance [7,8,9]. Lipids are a primary energy source and essential structural components of cellular membranes [10]. Lipid metabolism is a dynamic process, with tumor tissues undergoing lipid remodeling via key lipid-metabolizing enzymes, transcription factors, and signaling pathways [11,12,13]. Studies have revealed that lipid metabolism and associated genes (LMRGs) play a critical role in LUAD development and prognosis [14,15].

Prostaglandin D synthase (PTGDS) is a lipid metabolism-related gene on chromosome 9. PTGDS belongs to the lipocalin superfamily and is involved in prostaglandin metabolism and lipid transport [16]. Interestingly, PTGDS plays varying roles across different types of cancer. It promotes tumor progression in diffuse large B-cell lymphoma, hepatocellular adenoma, testicular cancer, and ovarian cancer [17,18,19,20], whereas its downregulation in prostate, lung, and gastric cancers is associated with poor prognosis [21,22,23]. Hence, the role of PTGDS in LUAD progression requires further investigation.

However, the functional role and molecular mechanism of PTGDS in LUAD progression remain unclear. In this study, we integrated bioinformatics and experimental approaches. We first analyzed PTGDS expression, its prognostic significance, and its associations with cell cycle regulators, immune cell infiltration, and immune checkpoint genes in LUAD and then extended this analysis across multiple cancer types. We then experimentally validated that PTGDS suppresses LUAD cell proliferation, migration, and invasion in vitro and in vivo. These findings suggest that PTGDS may exert a tumor-suppressive effect in LUAD by modulating cell cycle regulation and the immune microenvironment, positioning it as a potential prognostic biomarker and therapeutic target for LUAD.

## 2. Materials and Methods

### 2.1. Data Collection

The gene expression data for the differential analysis of non-small cell lung cancer (NSCLC) were sourced from the Gene Expression Omnibus (GEO) database (http://www.ncbi.nlm.nih.gov/geo/, accessed on 4 June 2026) with the accession number GSE118370. A dataset comprising 743 genes associated with lipid metabolism was obtained from the GeneCards website (https://www.genecards.org/, accessed on 4 June 2026). Gene expression data and clinical information for the prognosis analysis of lung adenocarcinoma (LUAD) were retrieved from The Cancer Genome Atlas (TCGA) database (https://portal.gdc.cancer.gov/repository, accessed on 4 June 2026). The clinical characteristics and genetic variation data of patients with LUAD were obtained from the TIMER2.0 database (http://timer.cistrome.org/, accessed on 4 June 2026). For pan-cancer analysis, standardized pan-cancer datasets and PTGDS expression data were retrieved from the UCSC Xena database (https://xenabrowser.net/, accessed on 4 June 2026). Pan-cancer clinical relevance and immune infiltration scores were calculated using the TISIDB database (http://cis.hku.hk/TISIDB/, accessed on 4 June 2026). Immune cell fractions were examined using the CIBERSORT algorithm (via the immunedeconvR package). Potential immunotherapy responses were predicted using the TIDE (Tumor Immune Dysfunction and Exclusion) algorithm.

### 2.2. Identification of Differentially Expressed Lipid Metabolism-Related Genes

The R package “limma” was used to analyze genes in LUAD from TCGA and the GSE118370 dataset. Genes with an FDR < 0.05 and |FoldChange| > 1.5 were identified as differentially expressed. A Venn diagram (https://hiplot-academic.com/basic/Venn, accessed on 4 June 2026) was used to identify differentially expressed lipid metabolism-related genes by overlapping the two sets with the lipid metabolism-related gene set.

### 2.3. DNA Methylation Analysis

DNA methylation data of PTGDS in LUAD were obtained from the TCGA database. Methylation levels (beta-values) were compared between tumor and adjacent normal tissues using the two-sided Wilcoxon rank-sum test.

### 2.4. PTGDS Co-Expression Analysis and Functional Enrichment of Differentially Expressed PTGDS-Related Genes

Pearson’s correlation coefficient was used to determine the co-expression between PTGDS expression and other genes. To define high and low PTGDS expression groups in TCGA-LUAD samples, the median expression value of PTGDS was used as the cutoff. The R package “limma” was used to analyze gene expression in tumor and adjacent non-tumor tissues from TCGA data of patients with LUAD, categorizing PTGDS expression into high and low groups. Genes with an FDR < 0.05 and |FoldChange| > 1.5 were identified as PTGDS-related differential genes. Differential genes were analyzed for gene ontology (GO) and Kyoto Encyclopedia of Genes and Genomes (KEGG) pathway enrichment. Statistically significant differences (*p*-value < 0.05) were visualized using the Cairo and ggplot2 packages of R software (v4.1.0).

### 2.5. Immune Microenvironment Assessment

The R software was used to estimate the stromal, immune, and ESTIMATE scores for LUAD. The R package CIBERSORT was used to examine the proportions of 22 common immune cell types. To perform reliable immune score evaluation, we employed the immunedeconvR package and utilized the ggClusterNet R package for analysis and visualization of the results. We applied the TIDE (Tumor Immune Dysfunction and Exclusion) algorithm to predict potential immunotherapy responses.

### 2.6. Pan-Cancer Analysis of PTGDS

The standardized pan-cancer dataset was retrieved from the UCSC database (https://xenabrowser.net/, accessed on 4 June 2026). The expression data of the PTGDS gene in various samples were also extracted. R software was used to estimate the differences in expression between normal and tumor samples according to cancer type, using an unpaired Student’s t-test for significance assessment. The coxph function from the R package survival was used to construct a Cox proportional hazards regression model, examining the correlation between gene expression and prognosis across various cancer types using the Logrank test for statistical significance. Additionally, the clinical relevance of PTGDS across pan-cancer was calculated using the TISIDB database (http://cis.hku.hk/TISIDB/, accessed on 4 June 2026). The infiltration scores of T cells, CD8+ T cells, cytotoxic lymphocytes, B lineage, NK cells, monocytic lineage, myeloid dendritic cells, neutrophils, endothelial cells, and fibroblasts in each cancer type were reassessed based on gene expression. The tumor mutational burden (TMB) scores were determined for each cancer type from published studies [24], and the Pearson correlation was calculated.

### 2.7. Collection of LUAD Samples

Between 2015 and 2018, 31 pairs of LUAD and matched adjacent normal lung tissues were collected from surgeries performed at the National Cancer Center/National Clinical Research Center for Cancer/Cancer Hospital, Chinese Academy of Medical Sciences, and Peking Union Medical College. The collection of these specimens was approved by the Medical Ethics Committee of the National Cancer Center/National Clinical Research Center for Cancer/Cancer Hospital, Chinese Academy of Medical Sciences, and Peking Union Medical College.

### 2.8. Cell Culture and Transfection

Human LUAD cell lines (H1299, A549 and H1944) were obtained from the Chinese Academy of Sciences (Beijing, China). H1299 and H1944 cells were cultured in RPMI-1640 medium (Gibco, Grand Island, New York, NY, USA) supplemented with 10% FBS (Pricella, Wuhan, China) and 1% penicillin-streptomycin (Beyotime, Shanghai, China). A549 cells were cultured in 10 cm dishes (Corning, New York, NY, USA) with DMEM (Gibco, Grand Island, New York, NY, USA) supplemented with 10% FBS (Pricella, Wuhan, China) and 1% penicillin-streptomycin (Beyotime). Both cell lines were cultured at 37 °C under a 5% CO_2_ atmosphere. PTGDS plasmids (General Biol, Chuzhou, China) were packaged into lentivirus and used to infect H1299 and A549 cells. Stable transductants were selected with puromycin for two weeks. For transient PTGDS knockdown, siRNA targeting PTGDS was transfected using Lipofectamine 8000. The efficiency of both stable overexpression and transient knockdown was confirmed by Western blot.

### 2.9. Quantitative Reverse Transcription Polymerase Chain Reaction (qRT-PCR)

RNA was extracted from tissues, and cells were lysed with TRIzol reagent (Invitrogen, Carlsbad, CA, USA), followed by precipitation and washing with chloroform, isopropanol, and 75% ethanol. The RNA pellet was dissolved in 30 µL of deionized water, and its concentration was measured for further analysis. Subsequently, cDNA was synthesized using HiScript II Q RT SuperMix for qPCR. Then, cDNA was analyzed by qRT–PCR using AceQ qPCR SYBR Green Master Mix (without ROX) (Vazyme, Nanjing, China) following the manufacturer’s protocol. The primers used were GAPDH (Forward: 5′-GGAGCGAGATCCCTCCAAAAT-3′, Reverse: 5′-GGCTGTTGTCATACTTCTCATGG-3′) and PTGDS (Forward: 5′-GGCGTTGTCCATGTGCAAG-3′, Reverse: 5′-GGACTCCGGTAGCTGTAGGA-3′).

### 2.10. Immunohistochemistry (IHC)

The paraffin sections were baked, deparaffinized, hydrated, and washed. Antigen retrieval was performed, and peroxidase activity was inhibited. The sections were blocked with an antigen-blocking solution and incubated overnight at 4 °C with a polyclonal rabbit anti-PTGDS antibody (1:500, Proteintech, Wuhan, China). The sections were then incubated with a secondary antibody (1:1000, Huabio, Hangzhou, China) at room temperature for 2 h. Staining was performed using DAB and hematoxylin.

### 2.11. Immunohistochemistry (IHC) Scoring

The stained sections were independently evaluated by two pathologists who were blinded to the clinical and outcome data. PTGDS expression was assessed using a semi-quantitative scoring system combining staining intensity (0, no staining; 1, weak; 2, moderate; 3, strong) and the percentage of positive tumor cells (0–100%). The final IHC score was calculated as intensity × percentage (range 0–300). The cutpoint for “high” versus “low” PTGDS expression was prespecified as the median IHC score of the 31 tumor samples to ensure consistency with the TCGA analysis. Inter-observer discrepancies were resolved by consensus.

### 2.12. Western Blot (WB)

Tissues and cells were lysed using RIPA buffer containing protease and phosphatase inhibitors (CWbio, Taizhou, China), then boiled for 10 min to denature. SDS-PAGE was performed on 20 µg of protein and 2.5 µL of pre-stained protein marker (Vazyme, Nanjing, China) and subsequently transferred to a PVDF membrane (Millipore). The membrane was blocked with 5% non-fat milk for 1 h at room temperature, followed by overnight incubation at 4 °C with the primary antibody. Subsequently, the membrane was incubated with a goat anti-rabbit secondary antibody for 1 h. Images were captured using an enhanced chemiluminescence reagent (Vazyme, Nanjing, China) and an imaging system.

The antibodies used were PTGDS (1:1000, Proteintech, Wuhan, China), GAPDH (1:100,000, Huabio, Hangzhou, China), β-Actin (1:10,000, Huabio, Hangzhou, China), CDK1 (1:2000, Huabio, Hangzhou, China), p21 (1:1000, Proteintech, Wuhan, China), p27 (1:1000, Proteintech, Wuhan, China) and PLK1 (1:1000, Immunoway, Plano, TX, USA).

### 2.13. Immunofluorescence

LUAD cells subjected to different treatments were fixed on coverslips with 4% paraformaldehyde. Cells were treated with 0.1% Triton X-100 for permeabilization and blocked with 10% goat serum for 30 min at room temperature. After blocking, the cells were incubated at 4 °C overnight with either rabbit anti-human CDK1 (1:100, Huabio, Hangzhou, China) or rabbit anti-human PLK1 (1:200, Immunoway, Plano, TX, USA). The cells were incubated in the dark with iFluor 594-labeled goat anti-rabbit IgG (1:500, Huabio, Hangzhou, China) for 60 min, followed by DAPI staining of the nuclei for 5 min (Beyotime, Shanghai, China). Finally, the cells were examined under a confocal microscope at 200× magnification.

### 2.14. Cell Counting Kit-8 (CCK-8) Assay

LUAD cells subjected to different treatments were seeded into 96-well plates. Then, 10 µL of CCK-8 reagent (APE × BIO, Houston, Texas, USA) was added to each well and incubated for 2 h at 24, 48, or 72 h. The absorbance of each well was measured at 450 nm using a multifunctional microplate reader. All experiments were performed with three independent biological replicates (*n* = 3).

### 2.15. Colony Formation Assay

LUAD cells were seeded into 6-well plates at a density of 1 × 10^3^ cells per well, following various treatments. The medium was replaced every three days. After 10 days of growth, the medium was discarded, and the cells were fixed with 4% paraformaldehyde and washed with PBS. The cells were stained with crystal violet for 10 min. Finally, the plates were air-dried in a ventilated area and photographed. Three independent biological replicates were performed.

### 2.16. EdU Assay

LUAD cells were seeded into 6-well plates after various treatments. EdU was added to the medium at 10 µM for 2 h after cell density reached 60–70%. The media were discarded, and the cells were fixed for 15 min in 4% paraformaldehyde before being permeabilized with 0.1% Triton X-100. Cells were stained with BeyoClick^TM^ EdU-555 (Beyotime, Shanghai, China) and Hoechst dye at room temperature in the dark. Finally, fluorescence was detected using a laser confocal microscope. Experiments were repeated three times independently.

### 2.17. Wound-Healing Assay

LUAD cells treated with various methods were seeded into 6-well plates until 90% confluence was achieved. A scratch was created by a 200 µL pipette tip in the cell monolayer. The cell scratch was examined under a microscope at 0 and 48 h. ImageJ software (1.54j) was used to estimate the scratch area. Three independent biological replicates were conducted, and the investigator performing the area measurement was blinded to group allocation.

### 2.18. Transwell Assay

The Transwell chambers were optionally coated with Matrigel. After various treatments, LUAD cells (2 × 10^4^ per well) were seeded into the upper chamber of 24-well Transwell inserts (Corning, New York, NY, USA) with serum-free media. The lower chamber was filled with 600 µL of medium containing FBS. After 24 h, the cells in the Transwell inserts were fixed with paraformaldehyde and stained with crystal violet. The images were captured using a microscope. Three independent biological replicates were performed, and cell counting was conducted by an investigator blinded to the treatment conditions.

### 2.19. Cell Cycle Analysis

H1299 and A549 cells were digested and transfected with PTGDS plasmids after 24 h of culture. The cells were washed with PBS and fixed overnight at 4 °C with 70% ethanol. After PBS washing, the cells were resuspended in a PI and RNase solution and analyzed via flow cytometry. FlowJo software (10.8) was used to analyze the data. Experiments were performed in triplicate (*n* = 3).

### 2.20. Establishment of the Nude Mouse Xenograft Tumor Model

A PTGDS xenograft model was developed using 4-week-old BALB/c nude mice at the Laidi Biomedical Research Institute. The Ethics Committee of the Laidi Biomedical Research Institute approved the animal study protocol. BALB/c nude mice (*n* = 5 per group) were subcutaneously injected with 2 × 10^6^ H1299 cells under various treatments. Tumor growth was monitored, and tumor size was determined using calipers every five days. Tumor volume was determined using the formula: V = (a × b^2^)/2. On day 25, the mice were euthanized, and the tumors were surgically removed. Tumor tissues were collected to determine their weight and volume, and proteins were extracted for subsequent analysis.

### 2.21. Statistical Analyses

Data were all analyzed using R software (version 4.1.0) with appropriate Bioconductor packages, as well as GraphPad Prism (version 8.0). For differential gene expression analysis, the limma package was used with the Benjamini–Hochberg (BH) method to adjust *p*-values, and |FoldChange| > 1.5 with adjusted *p*-value < 0.05 was considered statistically significant. For multiple group comparisons, one-way analysis of variance (ANOVA) followed by Tukey’s post hoc test was applied. Associations between PTGDS expression and clinicopathological characteristics were evaluated using the two-sided χ^2^ test or Fisher’s exact test as appropriate. Survival curves were generated using the Kaplan–Meier method and compared using the log-rank test. Univariate and multivariate Cox proportional hazards regression models were performed to assess the prognostic value of PTGDS. Results are presented as hazard ratios (HR) with 95% confidence intervals (CI). For all in vitro experiments, at least three independent biological replicates were performed (*n* = 3), and data are presented as mean ± standard deviation (SD). A *p*-value < 0.05 was considered statistically significant for all analyses unless otherwise specified.

## 3. Results

### 3.1. Downregulation of the Lipid Metabolism-Related Gene PTGDS in LUAD and Its Positive Correlation with Prognosis

**Figure 1:**
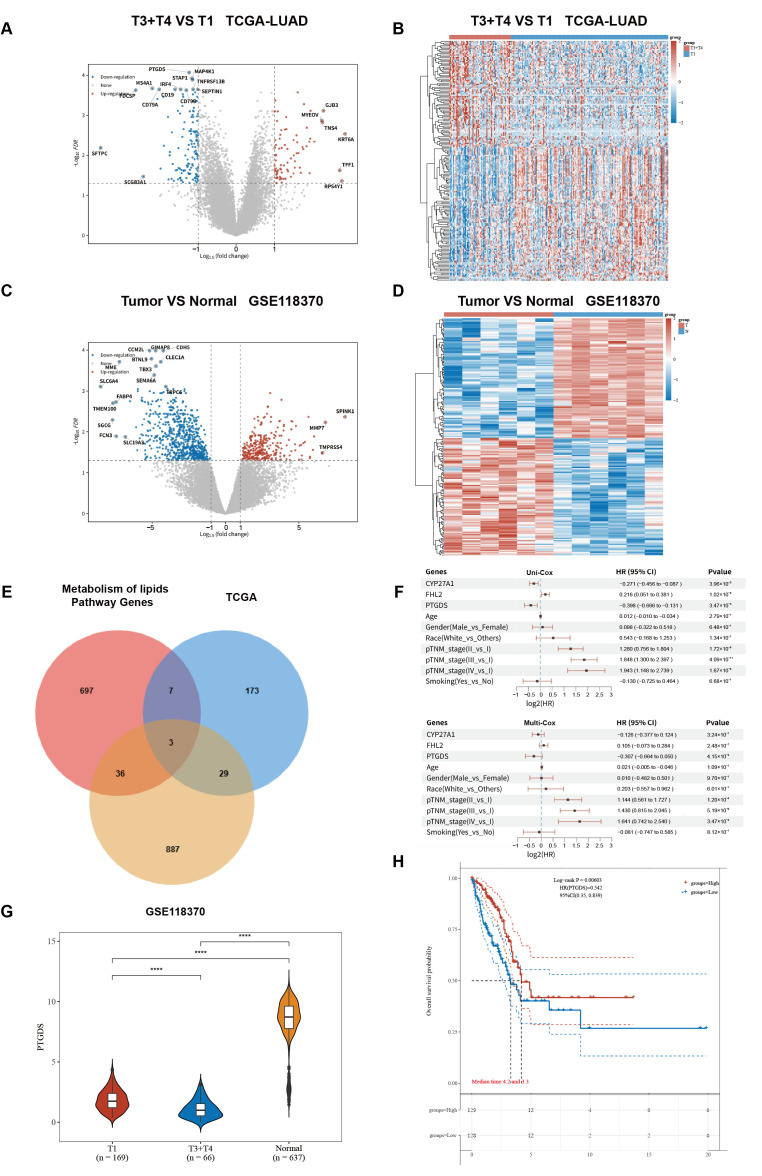
(**A**). Volcano plot of differential genes between T3 + T4 and T1 LUAD in TCGA. (**B**). Heatmap of differential genes between T3 + T4 LUAD and T1 LUAD in TCGA. (**C**). Volcano plot of differential genes between LUAD tumors and adjacent normal tissues in the GEO dataset (GSE118370). (**D**). Heatmap of differential genes between LUAD tumors and adjacent normal tissues in the GEO dataset (GSE118370). (**E**). Venn diagram of differentially expressed genes and lipid metabolism-related genes. (**F**). Univariable Cox regression analysis and multivariable Cox regression analysis of FHL2, PTGDS, and CYP27A1 in LUAD. (**G**). Expression of PTGDS in different stages of LUAD and adjacent normal tissues. (**H**). Analysis of PTGDS expression and its association with LUAD prognosis. (**** *p* < 0.0001).

To investigate the role of lipid metabolism-related genes in the progression of lung adenocarcinoma (LUAD), we analyzed LUAD data from the TCGA cohort. By comparing differentially expressed genes (DEGs) between T3 + T4 stage and T1 stage LUAD patients, we identified 212 DEGs, including 79 upregulated and 133 downregulated genes in the T3 + T4 group (Figure 1A,B). Additionally, using sequencing data from 6 paired LUAD tumor and adjacent normal tissues in the GEO dataset GSE118370, we identified 955 DEGs, with 286 upregulated and 669 downregulated in tumors (Figure 1C,D). From the GeneCards database, we obtained a lipid metabolism-related gene set comprising 743 genes. Intersection analysis between these DEGs and lipid metabolism-related genes revealed 3 overlapping differentially expressed lipid metabolism-related genes (Figure 1E). Subsequently, we performed univariate COX regression analysis to assess the prognostic significance of these 3 genes. The results indicated that PTGDS and CYP27A1 were protective factors, while FHL2 was a risk factor in LUAD. The multivariate COX showed that PTGDS remained a protective factor. (Figure 1F). Given that CYP27A1 and FHL2 have been extensively studied in LUAD, we focused on PTGDS for further investigation. TCGA expression analysis showed that PTGDS was downregulated in tumor tissues, with even lower expression in higher T stages (Figure 1G). Prognostic analysis further revealed that PTGDS expression was negatively correlated with poor survival in LUAD patients (Figure 1H). These findings suggest that low PTGDS expression is closely associated with unfavorable progression in LUAD.

### 3.2. Clinical and Genetic Characteristics of the Lipid Metabolism-Related Gene PTGDS in LUAD

**Figure 2:**
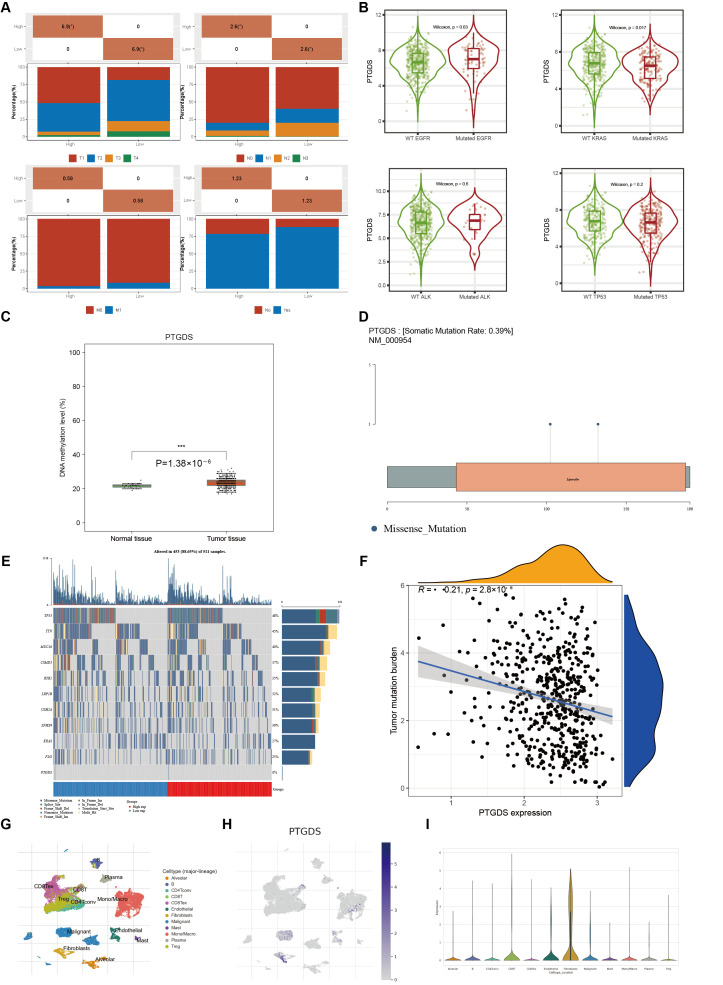
(**A**). Correlation between PTGDS expression and clinical features of LUAD in TCGA (* *p* < 0.05). (**B**). Expression of PTGDS in patients with LUAD harboring common gene mutations (EGFR, ALK, KRAS and TP53). (**C**). Expression of PTGDS in patients with LUAD harboring common gene mutations (EGFR, ALK, KRAS and TP53). (**D**). Mutation profile of PTGDS in LUAD from TCGA. (**E**). Mutational landscape of LUAD stratified by PTGDS expression levels. (**F**). Correlation between PTGDS expression and tumor mutational burden in LUAD. (**G**–**I**). Single-cell analysis of PTGDS expression distribution in lung cancer. (*** *p* < 0.001).

We conducted a comprehensive analysis of clinical data from LUAD patients in the TCGA database stratified by PTGDS expression levels. Our findings revealed significant differences in T-stage and N-stage distribution between LUAD with high PTGDS expression and those with low expression, while no significant variations were observed in M-stage or smoking status (Figure 2A). To further characterize PTGDS expression patterns in lung adenocarcinoma, we assessed its levels in the context of common driver mutations (EGFR, ALK, KRAS, TP53) using the TIMER2.0 database. This analysis revealed that PTGDS expression was significantly upregulated in tumors harboring EGFR mutations and downregulated in those with KRAS mutations (Figure 2B). Our analysis of PTGDS methylation status revealed significantly lower methylation levels in adjacent normal tissues compared to tumor tissues, providing a partial explanation for the higher PTGDS expression observed in normal adjacent tissue (Figure 2C). Further investigation of PTGDS mutation profiles in LUAD showed a low mutation rate (0.39%), with all mutations being nonsense variants (Figure 2D). Comparative analysis of the top 10 mutated genes between high and low PTGDS expression groups showed no significant differences, with TP53, TTN, and MUC16 being predominant in both groups (Figure 2E). Subsequent analysis revealed a negative correlation between PTGDS expression and tumor mutation burden in lung adenocarcinoma (Figure 2F). Single-cell RNA sequencing analysis using the EMTAB6149 dataset from the TISCH2 database demonstrated predominant PTGDS expression in fibroblasts, malignant cells, endothelial cells, and monocytes (Figure 2G–I).

### 3.3. Co-Expression Gene Analysis and Differential Gene Enrichment Analysis of the Lipid Metabolism-Related Gene PTGDS

**Figure 3:**
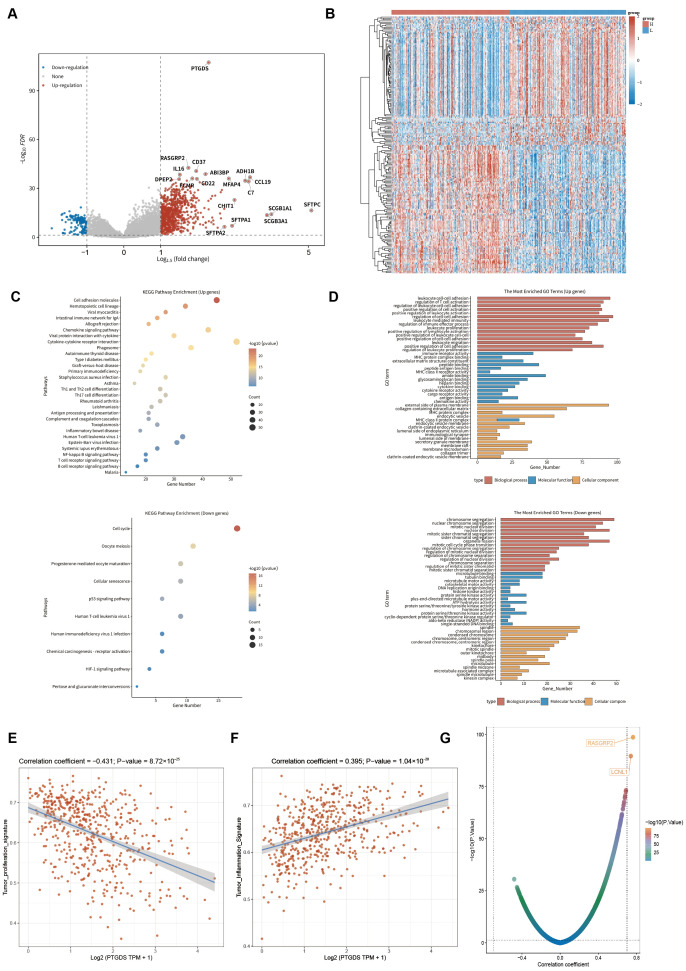
(**A**). Volcano plot of differentially expressed genes between PTGDS-high and PTGDS-low groups in TCGA-LUAD. (**B**). Heatmap of differentially expressed genes between PTGDS-high and PTGDS-low groups in TCGA-LUAD. (**C**). KEGG pathway enrichment results of differential genes. (**D**). GO pathway enrichment results of differential genes. (**E**). Correlation analysis between PTGDS expression and tumor proliferation-related gene expression in LUAD. (**F**). Correlation analysis between PTGDS expression and tumor inflammation-related gene expression in LUAD. (**G**). Volcano plot of correlation coefficients between PTGDS and all genes.

Subsequently, utilizing TCGA-LUAD data, we stratified patients into high and low PTGDS expression groups and performed differential gene expression analysis. Using a threshold of FDR < 0.05 and |FoldChange| > 1.5, we identified a total of 932 differentially expressed genes (DEGs), comprising 787 upregulated genes and 145 downregulated genes (Figure 3A,B). We conducted GO, KEGG pathway, and GSEA analyses to investigate the pathways and functions associated with PTGDS. Enrichment analysis demonstrated that differentially expressed genes were mainly involved in cell cycle and immune-related pathways (Figure 3C,D and Figure S1). Further analysis revealed significant correlations between PTGDS expression and key tumorigenic processes: PTGDS expression exhibited a negative correlation with the expression of tumor proliferation-associated genes while demonstrating a positive correlation with the expression of tumor immunity-associated genes (Figure 3E,F). Additionally, correlation analysis of LUAD patient expression data from the TCGA database identified two genes strongly co-expressed with PTGDS (R > 0.7): RASGRP2 and LCNL1 (Figure 3G).

### 3.4. Association Between the Lipid Metabolism-Related Gene PTGDS and Tumor Immune Microenvironment

**Figure 4:**
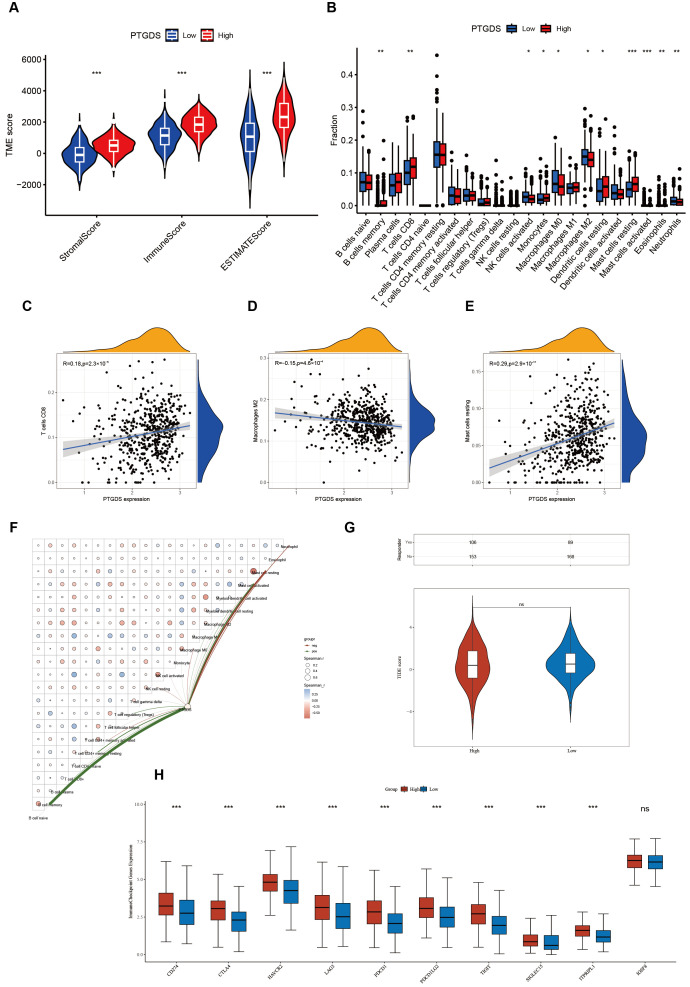
(**A**). Correlation between PTGDS expression and immune-related scores (StromalScore, ImmuneScore, and ESTIMATEScore) in patients with LUAD. (**B**). Relationship between PTGDS expression and the composition of immune cell infiltration in patients with LUAD. (**C**–**E**). Association of PTGDS expression with CD8+ T-cell, M2 macrophage and mast cell infiltration in patients with LUAD. (**F**). Heatmap of correlation between PTGDS expression and immune cell infiltration in LUAD. (**G**). Differential analysis of immunotherapy response between PTGDS-high and PTGDS-low expression groups. (**H**). Differential expression analysis of immune checkpoint genes between PTGDS-high and PTGDS-low groups. (* *p* < 0.05, ** *p* < 0.01, *** *p* < 0.001).

Enrichment analysis indicated the role of PTGDS in immune pathways, prompting further investigation into its relationship with immune infiltration. Bioinformatic analysis of the tumor microenvironment (TME) using the ESTIMATE algorithm revealed a positive association between high PTGDS expression and immune infiltration scores (Figure 4A), suggesting a potential link between PTGDS and immune cell abundance that warrants experimental validation. This study analyzed the variations in immune cell components between LUAD samples exhibiting high and low PTGDS expression (Figure 4B). LUAD samples with high PTGDS expression revealed an increased infiltration of CD8+ T cells and mast cells and a reduced proportion of M2 macrophages (Figure 4C–E). Further correlation analysis demonstrated that the infiltration of resting mast cells, CD8+ T cells, memory B cells, monocytes, and resting dendritic cells was positively associated with PTGDS expression. In contrast, activated NK cells, activated dendritic cells, M0 macrophages, M2 macrophages, eosinophils, activated mast cells, and neutrophils were negatively associated with PTGDS expression (Figure 4F). Subsequently, we analyzed differences in immune response between the high and low PTGDS expression strata. The results revealed no statistically significant differences in immune response between these groups (Figure 4G). However, PTGDS expression exhibited a positive correlation with the expression of the majority of immune checkpoint-related genes (Figure 4H).

### 3.5. Pan-Cancer Analysis of Lipid Metabolism-Related Gene PTGDS

**Figure 5:**
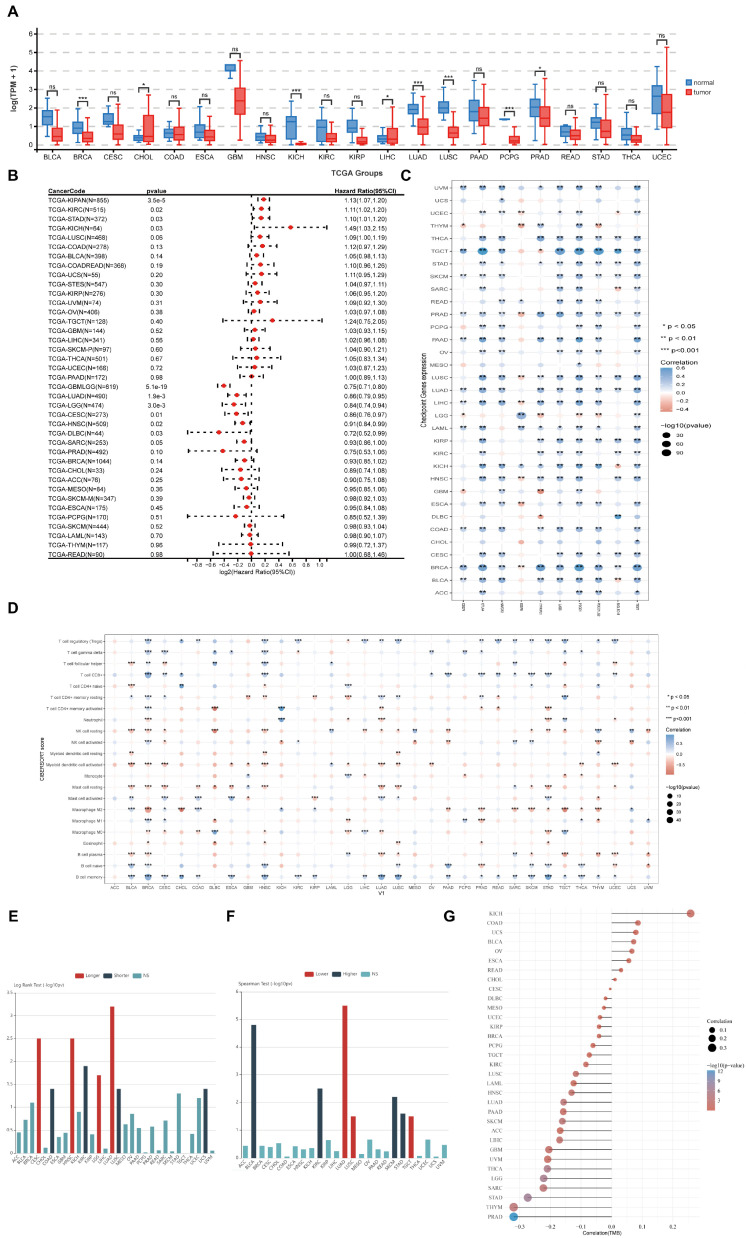
(**A**). Pan-cancer analysis of PTGDS expression. (**B**). Univariate risk model of PTGDS and prognosis across cancers. (**C**). Correlation between PTGDS expression and immune checkpoint-related genes in pan-cancer. (**D**). Correlation between PTGDS expression and immune cell infiltration in pan-cancer. (**E**). Association between PTGDS expression and OS across different cancers. (**F**). Correlation between PTGDS expression and clinical staging in pan-cancer analysis. (**G**). Bar plot of PTGDS expression correlation with tumor mutational burden (TMB) across pan-cancer. (* *p* < 0.05, ** *p* < 0.01, *** *p* < 0.001).

According to the literature, PTGDS is associated with various cancers. PTGDS expression was analyzed using RNA sequencing data from the TCGA database for diverse cancer types and normal tissues. The results indicated that PTGDS is downregulated in BRCA, KICH, LIHC, LUAD, LUSC, PCPG, and PRAD, while it is upregulated in CHOL (Figure 5A). Univariate Cox regression analysis was used to evaluate the prognostic significance of PTGDS in pan-cancer. The results revealed that PTGDS serves as a risk factor for KIPAN, KIRC, STAD, and KICH while acting as a protective factor in GBMLGG, LUAD, LGG, CESC, HNSC, and DLBC (Figure 5B). Analysis across multiple tumor types revealed that PTGDS expression positively correlated with most immune checkpoint genes (Figure 5C). Further investigation indicated a positive correlation between PTGDS expression and immune cell infiltration in various cancers, notably with CD8 T cells in BRCA, HNSC, and PAAD (Figure 5D). Subsequently, the clinical relevance of PTGDS was explored for various cancers using the TISIDB database. Higher PTGDS expression was correlated with longer overall survival (OS) in patients with CESC, HNSC, LGG, and LUAD but with shorter OS in patients with COAD, KIRC, LUSC, and UCS (Figure 5E). Higher PTGDS expression was associated with earlier clinical stages in LUAD, LUSC, and TGCT patients, whereas it was associated with later clinical stages in BLCA, KIRC, SKCM, and STAD patients (Figure 5F). The association between PTGDS and TMB across various cancer types was also investigated. PTGDS exhibited a significant association with MSI in nine cancers, revealing a positive correlation in KICH and a negative correlation in GBM, UVM, THCA, LGG, SARC, STAD, THYM and PRAD (Figure 5G).

### 3.6. Lipid Metabolism-Related Gene PTGDS Is Downregulated in LUAD and Correlates with Prognosis

**Figure 6:**
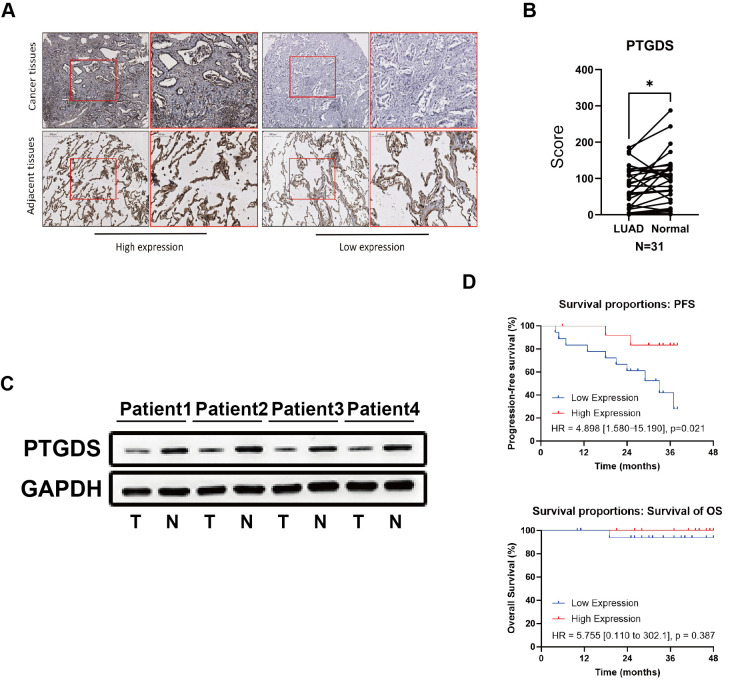
(**A**). IHC staining of PTGDS in tumor and adjacent normal tissues of patients with LUAD. (**B**). IHC scoring of PTGDS was performed on tumor tissues and paired adjacent normal tissues from 31 patients with LUAD. (**C**). WB analysis of PTGDS expression in tumor tissues versus adjacent normal tissues in patients with LUAD. (**D**). Correlation between PTGDS expression and progression-free survival and OS in patients with LUAD. (*: *p* < 0.05).

Tumors and adjacent normal tissues from 31 patients with LUAD were collected and subjected to IHC staining to detect PTGDS. PTGDS was significantly expressed in normal tissues but was decreased in tumor tissues (Figure 6A,B). WB analysis further validated this finding (Figure 6C). The clinical, pathological, and prognosis data revealed a significant association between low PTGDS expression and larger tumor diameter and advanced T stage in patients with LUAD (Table 1). Kaplan–Meier survival analysis indicated that elevated PTGDS expression correlated with extended disease-free survival and improved prognoses (Figure 6D).

### 3.7. Lipid Metabolism-Related Gene PTGDS Suppresses Proliferation, Migration, and Invasion of LUAD Cells In Vitro

**Figure 7:**
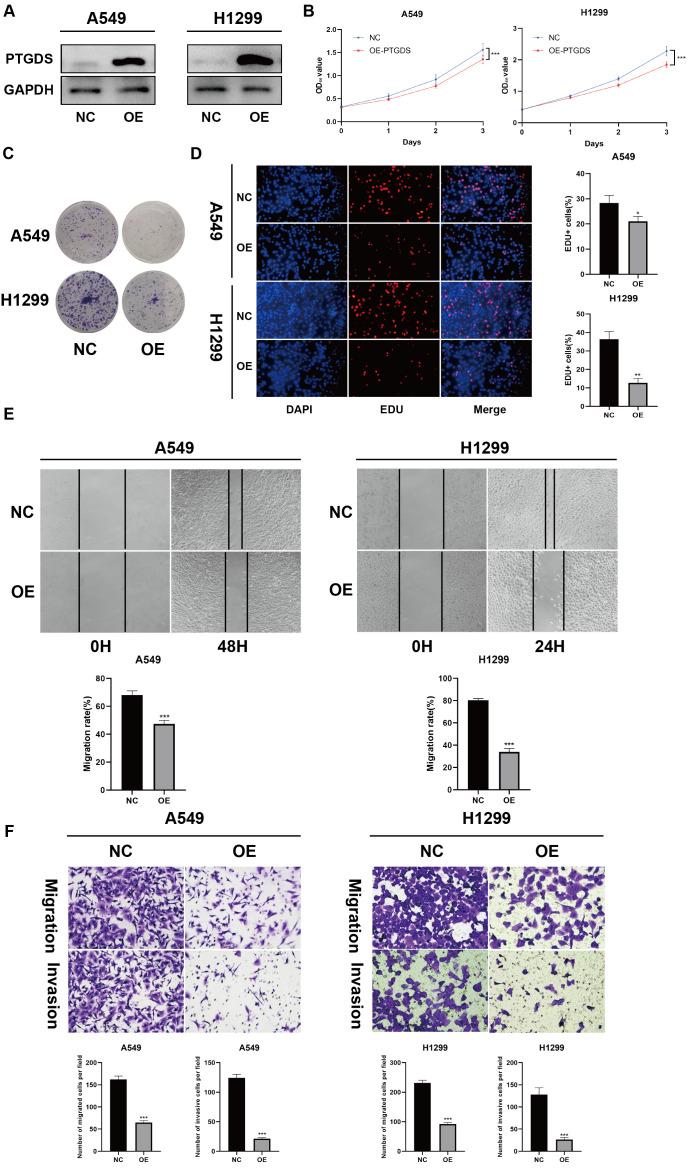
(**A**). Analysis of PTGDS overexpression in A549 and H1299 LUAD cells. (**B**). CCK-8 assay to evaluate the proliferation of NC and OE A549 and H1299 cells. (**C**). Colony formation assay in NC, PTGDS-OE A549, and H1299 cells. Colonies were counted after crystal violet staining at 10 days (stable overexpression). (**D**). Quantification of EDU-positive cells in NC and PTGDS-OE A549 and H1299 cells. (**E**). Scratch wound-healing assay assessing the migration capacity of NC and PTGDS-OE A549 and H1299 cells. (**F**). Transwell assay evaluating the migration and invasion capabilities of NC and PTGDS-OE A549 and H1299 cells. (* *p* < 0.05, ** *p* < 0.01, *** *p* < 0.001).

We examined PTGDS expression in LUAD cell lines by PCR, and the results showed that PTGDS expression was low in A549 and H1299 cells but high in H1944 cells (Figure S2A). To further investigate the role of PTGDS, it was overexpressed in A549 and H1299 cells and knocked down in H1944 cells. (Figure S2B,D and Figure 7A). CCK-8 assays demonstrated a significant reduction in absorbance in A549 and H1299 cells overexpressing PTGDS. Conversely, CCK-8 assays in PTGDS-knockdown H1944 cells showed a significant increase in absorbance, suggesting that PTGDS inhibits LUAD cell proliferation (Figure 7B and Figure S2E). Colony formation assays also revealed that the number of colonies formed by PTGDS-overexpressing LUAD cells was significantly reduced. In contrast, PTGDS knockdown in H1944 cells significantly increased colony formation, further confirming the suppression effect of PTGDS on cell proliferation (Figure 7C and Figure S2F). The EdU assay identified a lower ratio of EdU-positive cells in the PTGDS-overexpressing group compared with the control group, and PTGDS knockdown increased the EdU-positive ratio in H1944 cells, supporting the anti-proliferative function of PTGDS in LUAD cells (Figure 7D and Figure S2G). Wound-healing assays revealed a significant decrease in the migratory capacity of A549 and H1299 cells after PTGDS overexpression (Figure 7E). Knockdown of PTGDS in H1944 cells significantly enhanced wound closure (Figure S2H). The Transwell assays further indicated a significant decrease in the number of invading and migrating cells in PTGDS-overexpressing A549 and H1299 cells (Figure 7F). In PTGDS-knockdown H1944 cells, the numbers of invading and migrating cells were significantly increased (Figure S2I). These results demonstrated that PTGDS exerts anti-tumor effects in LUAD by inhibiting proliferation, migration, and invasion in vitro.

### 3.8. Lipid Metabolism-Related Gene PTGDS Is Closely Associated with Cell Cycle-Related Proteins

**Figure 8:**
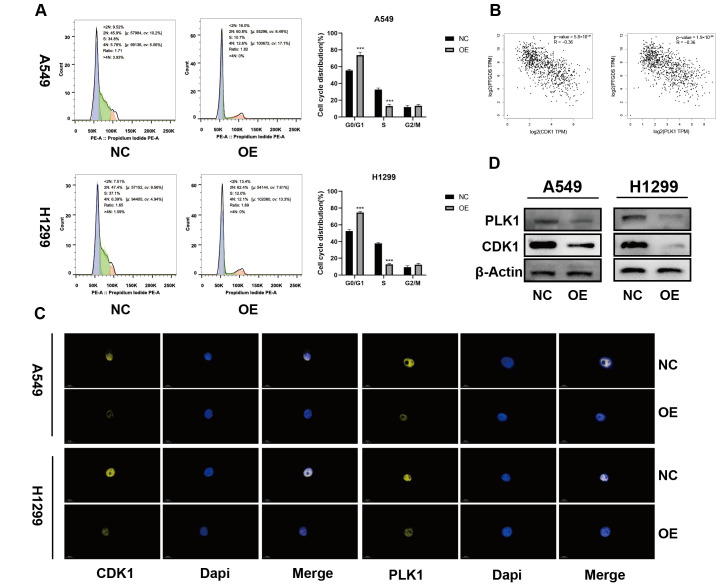
(**A**). Flow cytometry analysis of cell cycle alterations in NC and PTGDS-OE A549 and H1299 cells. (**B**). Correlation between PTGDS expression and the cell cycle-related proteins PLK1 and CDK1 in tumor and adjacent normal tissues of patients with LUAD. (**C**). Immunofluorescence analysis of PLK1 and CDK1 expression in NC and PTGDS-OE A549 and H1299 cells. (**D**). WB analysis of PLK1 and CDK1 expression in NC and PTGDS-OE A549 and H1299 cells. (***: *p* < 0.001).

Prior GO and KEGG enrichment analyses revealed a significant association between PTGDS and the cell cycle. This study used flow cytometry to examine the cell cycle of A549 and H1299 cells overexpressing PTGDS to elucidate the tumor-suppressive effects of PTGDS in LUAD. PTGDS upregulation increased the proportion of cells in the G0/G1 phase and decreased the proportion in the S phase (Figure 8A). To understand the impact of PTGDS on the cell cycle, this study examined the correlation between PTGDS and cell cycle-related proteins in patients with LUAD using data from the TCGA database. Analysis from the GEPIA2 website indicated a negative correlation between PTGDS expression and PLK1 and CDK1 in tumor and adjacent normal tissues (Figure 8B). Immunofluorescence and WB assays were performed to measure the expression of cell cycle-related proteins in the control and PTGDS-overexpressing groups (Figure 8C,D). We also examined the expression changes in the cell cycle inhibitors p21 and p27 proteins. Western blot results showed that p21 and p27 protein expression was increased in A549 and H1299 cells overexpressing PTGDS (Figure S3A). To further explore how PTGDS regulates the expression of cell cycle-related proteins, we measured the level of its downstream metabolite PGD2 using LC/MS. The results showed that PTGDS overexpression increased PGD2 production (Figure S3B). We then treated PTGDS-knockdown H1944 cells with exogenous PGD2 and examined the expression of cell cycle-related proteins by Western blot (Figure S3C). The results demonstrated that PGD2 supplementation reversed the changes in cell cycle protein expression induced by PTGDS knockdown. The findings indicated PTGDS is closely associated with cell cycle-related proteins.

### 3.9. Lipid Metabolism-Related Gene PTGDS Suppresses LUAD Progression In Vivo

**Figure 9:**
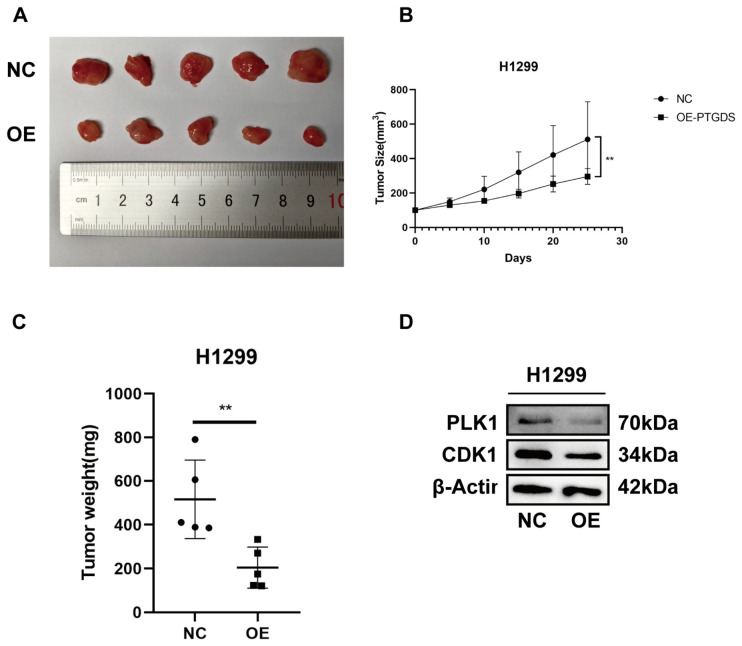
(**A**). Subcutaneous tumors from NC and PTGDS-OE H1299 cells in mice. (**B**,**C**). Tumor volume (**B**) and weight (**C**) comparison between NC and PTGDS-OE H1299 subcutaneous tumors. (**D**). WB analysis of PLK1 and CDK1 expression in NC and PTGDS-OE H1299 subcutaneous tumors. (**: *p* < 0.01).

To further investigate the role of PTGDS in vivo, xenograft experiments were performed using naive-control (NC) H1299 cells and H1299 cells stably overexpressing PTGDS. The results demonstrated that PTGDS significantly inhibited subcutaneous tumor growth in mice (Figure 9A). Tumor volume and mass measurements indicated that tumors in the OE-PTGDS group were significantly smaller compared to the NC group (Figure 9B,C). Subsequently, WB analysis was performed to detect cell cycle-related proteins, and the results revealed that PTGDS overexpression markedly reduced the expression of PLK1 and CDK1 and increased the expression of p21 and p27, providing a potential explanation for the tumor-suppressive effects of PTGDS (Figure 9D and Figure S3D).

## 4. Discussion

Despite numerous studies, LUAD remains a significant global health issue [1,25,26]. Metabolic abnormalities in tumors are a key cause of tumorigenesis and progression, with lipid metabolism playing an indispensable role [27]. Our previous systematic studies examined the involvement of lipid metabolism-related genes in LUAD and developed a risk-scoring model to predict patient prognosis and immunotherapy response [28].

Our integrated analysis demonstrates that the lipid metabolism gene PTGDS is significantly downregulated in LUAD tumor tissues compared to adjacent normal tissues, with reduced expression correlating strongly with advanced T stages, larger tumor size, lymph node involvement (N-stage), and poor patient survival—establishing it as a key protective factor in LUAD pathogenesis. This downregulation appears driven partly by epigenetic mechanisms, specifically hypermethylation in tumors, while genetic alterations like nonsense mutations are rare. Importantly, PTGDS expression shows distinct associations with driver mutations, being elevated in EGFR-mutant tumors but suppressed in KRAS-mutant contexts. Gene mutations are significant features of LUAD, and common mutations include EGFR, KRAS, ALK, ROS1, and RET [29,30]. Approximately 50% of Asians have activating EGFR mutations [31]. TMB is anticipated to produce more neoantigens, enhancing cell recognition, and has been clinically linked to improved outcomes with immune checkpoint inhibitors [32]. This study found that PTGDS is negatively correlated with TMB. Functional enrichment analysis of PTGDS in patients with LUAD revealed its primary enrichment in the cell cycle and immune infiltration pathways. Abnormalities in the cell cycle are an important cause of tumorigenesis, and the malignancy of tumors is often linked to an accelerated abnormal cell cycle [33,34,35]. Our data showed that PTGDS overexpression was associated with reduced expression of CDK1 and PLK1 and upregulated expression of p21 and p27, whereas PTGDS knockdown produced opposite effects. Flow cytometry further indicated that PTGDS overexpression increased the proportion of cells in the G0/G1 phase and decreased S-phase cells. These findings are consistent with a role for PTGDS in cell cycle regulation, but the precise molecular mechanism remains incompletely understood. Although we examined the expression of PGD2, a downstream metabolite of PTGDS, by LC/MS and preliminarily found that PGD2 levels can influence the expression of cell cycle proteins in LUAD, the specific molecular mechanism requires further investigation.

The immune microenvironment is another critical factor influencing tumor progression. The complex cellular cross-talk and signaling between tumor cells and immune microenvironment cells affect tumor progression [36,37,38]. In this study, we employed bioinformatic approaches to analyze the relationship between PTGDS and the immune microenvironment in LUAD, as well as its clinical significance. Immune cell infiltration in the tumor microenvironment (TME) is strongly linked to patient prognosis. Using computational methods (CIBERSORT, ESTIMATE, TIDE), we found a positive correlation between PTGDS expression and the infiltration of various immune cells, particularly an increased proportion of CD8+ T cells. Since PTGDS plays distinct roles in different tumors, exhibiting both tumor-suppressive and tumor-promoting effects, we conducted a preliminary pan-cancer analysis of PTGDS. The findings indicated that PTGDS is generally underexpressed in tumors and positively correlates with immune infiltration.

To further validate the tumor-suppressive role of PTGDS in LUAD, we verified PTGDS expression and its impact on prognosis using clinical patient tissue samples by Western blot and immunohistochemistry (IHC). Through the construction of LUAD cell lines with PTGDS overexpression and knockdown, followed by functional assays, we determined that PTGDS overexpression significantly inhibited LUAD cell proliferation, invasion, and migration, while PTGDS knockdown produced opposite effects, elucidating its role as a protective factor in LUAD patients. Flow cytometry analysis revealed that PTGDS overexpression increased the proportion of cells in the G0/G1 phase while decreasing the proportion in the S phase, consistent with the functional enrichment results related to the cell cycle pathway. Subsequently, we detected the expression of cell cycle-related proteins PLK1, CDK1, p21, and p27 by Western blot. The results showed that PTGDS inhibits LUAD progression by affecting the expression of cell cycle-related proteins. Finally, we established a mouse subcutaneous tumor model to validate the tumor-suppressive ability of PTGDS in vivo.

Several limitations of this study should be noted. First, our animal experiments only used PTGDS overexpression in nude mice, which lack a functional immune system. Therefore, we cannot directly test whether the immune-related findings from human data apply in vivo. Second, the patient tissue validation included only 31 pairs of samples, a relatively small number, and we could not perform multivariable survival analysis on this group. Third, the observed associations between PTGDS and cell cycle regulators are correlational. We have not yet proven a direct link, such as physical protein–protein interactions or involvement in gene transcriptional regulation. Fourth, all conclusions regarding the immune system are based on computational predictions (using tools such as CIBERSORT, ESTIMATE, and TIDE) and require experimental validation. To confirm whether PTGDS truly plays a role in LUAD progression and immune regulation, future studies will need to investigate PTGDS function in immunocompetent animal models and directly explore the underlying mechanisms. In summary, this study systematically integrates bioinformatics analysis with in vitro and in vivo functional validation, links PTGDS to cell cycle regulation, and provides preliminary evidence that exogenous PGD2 can reverse PTGDS knockdown-induced changes in cell cycle proteins. These findings offer new experimental evidence for the future development of PTGDS-targeted therapeutic approaches in LUAD patients and may help enrich treatment strategies for LUAD.

## 5. Conclusions

This study identified PTGDS, a lipid metabolism-related gene, as being downregulated in LUAD and highlighted its role as a protective factor positively associated with patient prognosis and immune infiltration. In vitro and in vivo experiments revealed that PTGDS inhibits LUAD cell proliferation, invasion, and migration by suppressing the expression of cell cycle-regulating proteins. The findings of this study indicate that PTGDS is a promising candidate biomarker and potential therapeutic target for LUAD, warranting further mechanistic and translational investigation.

## Figures and Tables

**Table 1:** Association between PTGDS expression and clinicopathological features in LUAD patients.

		Low Expression	High Expression	Total		*p*
gender					0.001	0.981
	female	11	8	19		
	male	7	5	12		
age					1.873	0.171
	≤60	10	4	14		
	>60	8	9	17		
grade					Fisher	0.237
	I–II	11	11	22		
	III	7	2	9		
diameter					Fisher	0.008
	>2 cm	10	1	11		
	≤2 cm	8	12	20		
TNM stage					Fisher	0.659
	I	15	11	26		
	II–III	3	2	5		
T classification					Fisher	0.010
	T1	10	13	23		
	T2–3	8	0	8		
N classification					Fisher	1.000
	N0	15	11	26		
	N1–3	3	2	5		
blood vessel invasion					Fisher	1.000
	no	14	11	25		
	yes	4	2	6		
Pleural invasion					Fisher	0.058
	no	13	13	26		
	yes	5	0	5		
Spread through air spaces					Fisher	1.000
	no	13	9	22		
	yes	5	4	9		

## Data Availability

The gene expression data for differential analysis of NSCLC were sourced from the Gene Expression Omnibus (GEO) database (http://www.ncbi.nlm.nih.gov/geo/, accessed on 4 June 2026) with the accession number GSE29249. A dataset comprising 743 genes associated with lipid metabolism was obtained from the GeneCards website (https://www.genecards.org/, accessed on 4 June 2026). Gene expression data, as well as clinical information for NSCLC prognosis analysis, were retrieved from The Cancer Genome Atlas (TCGA) database (https://portal.gdc.cancer.gov/repository, accessed on 4 June 2026). Clinical characteristics and genetic variation data of lung adenocarcinoma (LUAD) patients were obtained from the UALCAN (https://ualcan.path.uab.edu/, accessed on 4 June 2026) and TIMER2.0 (http://timer.cistrome.org/, accessed on 4 June 2026) databases.

## Supplementary Materials

The following supporting information can be downloaded at https://www.mdpi.com/article/10.3390/cancers18121884/s1, Figure S1: GSEA enrichment results; Figure S2: Lipid metabolism-related gene PTGDS suppresses proliferation, migration, and invasion of LUAD cells in vitro; Figure S3: Lipid metabolism-related gene PTGDS is closely associated with cell cycle-related proteins.
