# Supplementary material for: Unveiling Lipid Metabolism-Related Gene PTGDS: A Tumor Suppressor in Lung Adenocarcinoma with Therapeutic Potential
Source: Cancers (Basel). 2026 Jun 9;18(12):1884. doi: 10.3390/cancers18121884 (PMC13297109; doi:10.3390/cancers18121884)
Supplement: Supplementary file 1 [file cancers-18-01884-s001.zip › cancers-4250214-supplementary.pdf]

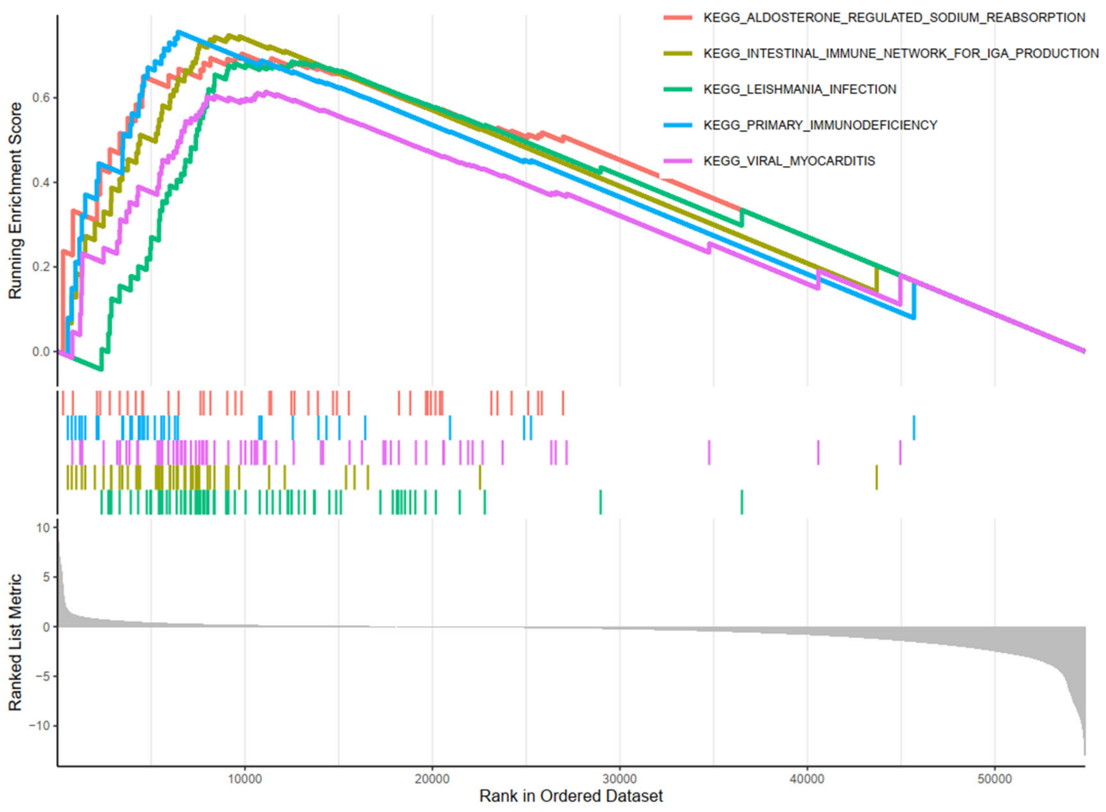

Figure S1: GSEA enrichment results

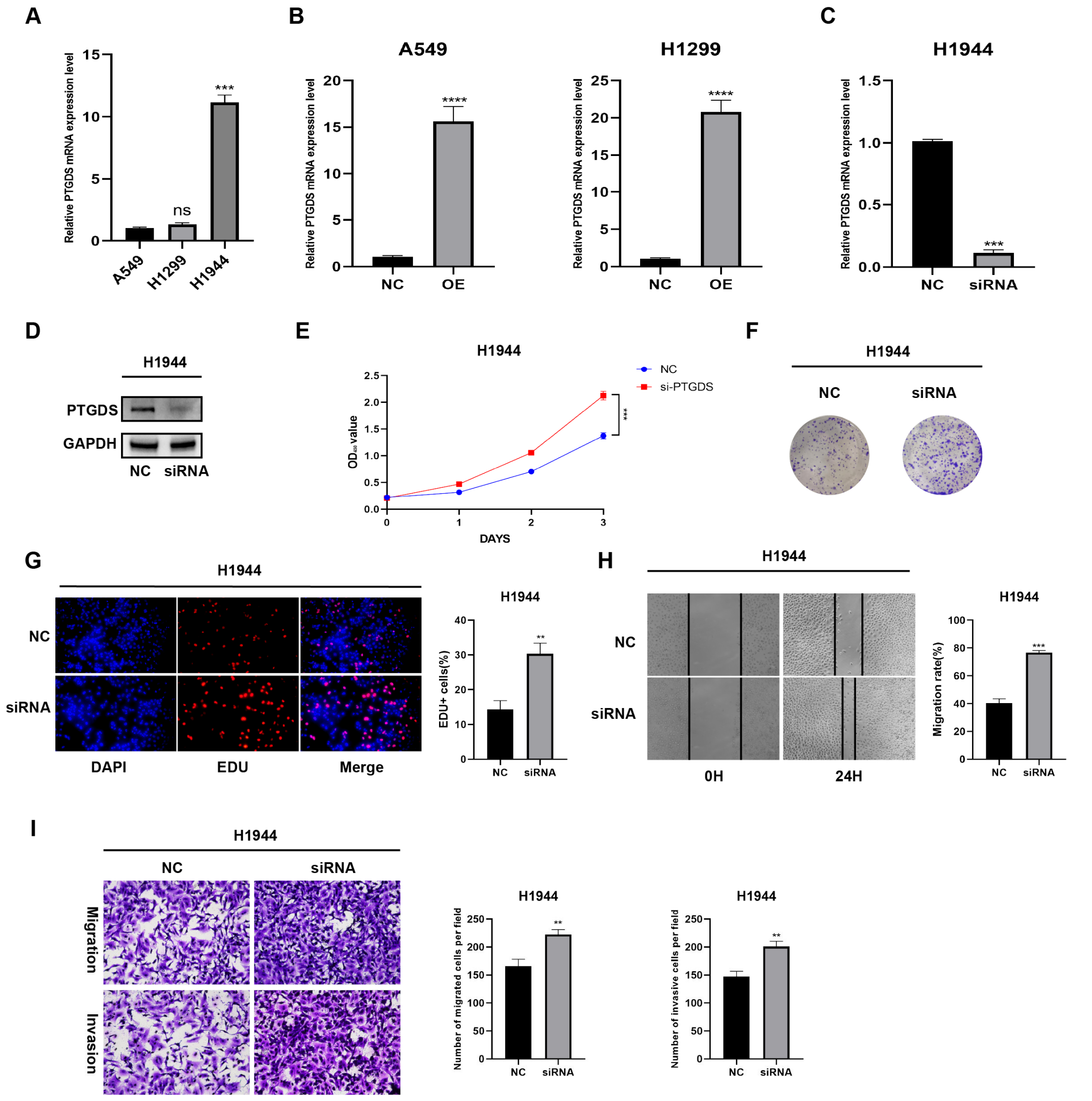

Figure S2: Lipid metabolism-related gene PTGDS suppresses proliferation, migration, and invasion of LUAD cells in vitro. A. qPCR detection of PTGDS expression in LUAD cells. B. qPCR detection of PTGDS expression in PTGDS-overexpressing A549 and H1299 cells. C. qPCR detection of PTGDS expression in si-PTGDS H1944 cells. D. Western blot detection of PTGDS expression in si-PTGDS H1944 cells. E. CCK-8 assay evaluating the proliferation of NC and si-PTGDS H1944 cells. F. Colony formation assay in NC and si-PTGDS H1944 cells; colonies were counted after crystal violet staining at 10 days (stable overexpression). G. Quantification of EdU-positive cells in NC and si-PTGDS H1944 cells. H. Scratch wound-healing assay assessing the migration capacity of NC and si-PTGDS H1944 cells. I. Transwell assay evaluating the migration and invasion capabilities of NC and si-PTGDS H1944 cells. (ns, not significant, ** $p < 0.01$, *** $p < 0.001$).

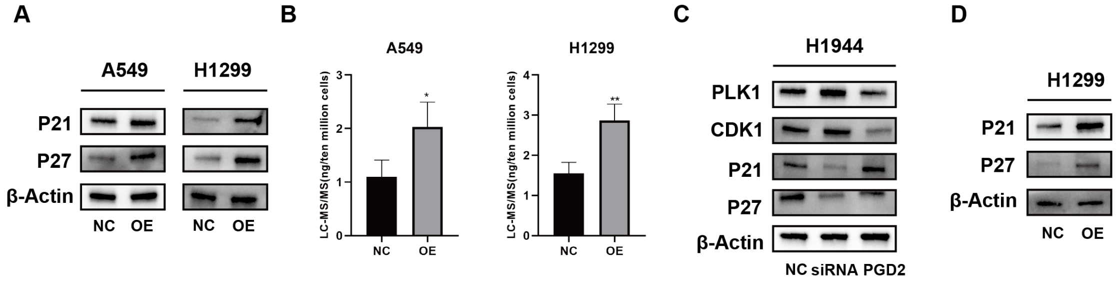

Figure S3: Lipid metabolism-related gene PTGDS is closely associated with cell cycle-related proteins. A. Western blot detection of p21 and p27 protein expression changes in A549 and H1299 cells. B. LC/MS detection of PGD2 levels in A549 and H1299 cells. C. Western blot detection of cell cycle-related protein expression changes in NC, si-PTGDS, and PTGDS-knockdown plus PGD2 supplementation H1944 cells. D. Western blot detection of p21 and p27 protein expression changes in H1299 subcutaneous xenograft tumors.
